# Scalable Semantic Adaptive Communication for Task Requirements in WSNs

**DOI:** 10.3390/s25092823

**Published:** 2025-04-30

**Authors:** Hong Yang, Xiaoqing Zhu, Jia Yang, Ji Li, Linbo Qing, Xiaohai He, Pingyu Wang

**Affiliations:** 1 College of Electronics and Information Engineering, Sichuan University, Chengdu 610065, China; 2 College of Communication Engineering, Chengdu University of Information Technology, Chengdu 610225, China

**Keywords:** adaptive sensing, scalable semantics, attention mechanism, task requirements

## Abstract

Wireless Sensor Networks (WSNs) have emerged as an efficient solution for numerous real-time applications, attributable to their compactness, cost effectiveness, and ease of deployment. The rapid advancement of the Internet of Things (IoT), Artificial Intelligence (AI), and sixth-generation mobile communication technology (6G) and Mobile Edge Computing (MEC) in recent years has catalyzed the transition towards large-scale deployment of WSN devices, and changed the image sensing and understanding to novel modes (such as machine-to-machine or human-to-machine interactions). However, the resulting data proliferation and the dynamics of communication environments introduce new challenges for WSN communication: (1) ensuring robust communication in adverse environments and (2) effectively alleviating bandwidth pressure from massive data transmission. To address these issues, this paper proposes a Scalable Semantic Adaptive Communication (SSAC) for task requirement. Firstly, we design an Attention Mechanism-based Joint Source Channel Coding (AMJSCC) in order to fully exploit the correlation among semantic features, channel conditions, and tasks. Then, a Prediction Scalable Semantic Generator (PSSG) is constructed to implement scalable semantics, allowing for flexible adjustments to achieve channel adaptation. The experimental results show that the proposed SSAC is more robust than traditional and other semantic communication algorithms in image classification tasks, and achieves scalable compression rates without sacrificing classification performance, while improving the bandwidth utilization of the communication system.

## 1. Introduction

Wireless Sensor Networks (WSNs) have emerged as highly effective solutions for a multitude of real-time applications owing to their compactness, cost effectiveness, and ease of deployment [1]. With the advancement of the Internet of Things (IoT) [2], Artificial Intelligence (AI) [2], and sixth-generation mobile communication technology (6G) [3], the sensing and understanding of image is changing to machine-to-machine or human-to-machine interactions. These developments present new challenges to the sensors and traditional communication technology. The emergence of semantic information can effectively address these challenges.

### 1.1. Backgrounds

In recent years, a new type of semantic communication [1,4,5,6] has become a new focus in the current research field, enabling a shift towards interaction between humans, machines, and objects. Task-oriented communication stands out as a disruptive technology for 6G system design by exploiting the task-specific information structures and folding the communication goals into the design of task-level transmission strategies [4]. The modern design of communication systems tends to adopt the Joint Source Channel Coding (JSCC) method [1], which integrates source characteristics and channel quality for joint optimization, thereby enhancing the transmission performance of the entire communication system. Compared to traditional communication systems that rely on independent source and channel encoding and decoding schemes, task-oriented end-to-end (E2E) semantic communication systems adopt different strategies to enable the receiver to execute specific tasks using the received semantic information. With the support of intelligent E2E communication, in [7], the authors studied a new semantic communication system framework, aiming to design a semantic source channel joint coding scheme while maximizing the capacity of the semantic communication system. Considering the dynamic communication environment with different background knowledge bases, the authors used transfer learning in [8] to jointly train semantic code and channel code. A semantic video conferencing is studied in [9] and a new wireless semantic transmission framework (Deep Video Semantic Transmission, DVST) is proposed in [10].

Task-oriented semantic communication has shown great potential in improving human–computer interaction efficiency and intelligent system task performance, as it can directly extract semantic information related to tasks for E2E transmission [11,12,13]. At present, the field of semantic communication is mainly divided into two categories based on the type of tasks at the receiver: data reconstruction and intelligent execution [14]. In [15], the authors proposed a universal E2E semantic communication model based on deep learning, including semantic code, channel code, and channel and background knowledge bases. The semantic code and channel code are implemented by deep neural networks (DNN). The authors proposed a semantic-based image retrieval system using semantic relationships between objects for different classification tasks [16]. Image classification tasks by semantic code and a task-oriented semantic communication system with foundation models are proposed in [17,18].

Scalable semantic communication for task requirements needs a multi-scale semantic feature extractor and the ability to resist the wireless noise. In [19], the authors proposed an Image Segmentation Semantic Communication (ISSC) system, which can extract the semantic features from the perceived images and transmit the features to the receiver that reconstructs the image segmentation. The authors proposed a semantic communication (SC) method with Artificial Intelligence Tasks (AITs), and named it SC-AITs [19] and then applied SC-AITs for an image classification task and to establish a prototype [20]. Experimental results show that SC-AITs have much lower bandwidth requirements and can achieve up to 40% classification accuracy gains compared with communications at the technical level. Some other teams are also researching semantic extraction and monitoring algorithms based on other tasks [21,22].

Semantic communication systems are still affected by physical noise in the communication environment. At present, most semantic communication systems for machine vision tasks are trained based on fixed channel conditions during the training phase [23], and the resulting training model is the best dedicated model for this SNR. If the channel quality is good and stable, the best prediction results can be obtained. However, in actual channels, there are effects such as multi-path fading, path loss, interference, and noise, and the channel quality will fluctuate within a certain range. Therefore, most existing task-oriented semantic communication systems commonly suffer from the problem of transmission rate adaptability under deteriorating channel quality. In order to achieve optimal communication performance, multiple training sessions can be conducted under different channel qualities to obtain multiple network models for switching usage in the event of channel quality fluctuations. The aforementioned issue consequently results in diminished computational efficiency, rendering the final task inadequately accomplished particularly in scenarios with limited computing resources.

In addition, semantic communication systems are also affected by semantic noise. To address this issue, in [24], the authors constructed a powerful end-to-end semantic communication framework using adversarial sample modeling methods, integrating samples containing semantic noise into the training set. The authors proposed a multi-task oriented semantic communication system that simultaneously considers the distortion of concise representation and semantic extension to address the problem of semantic noise in multi-task situations [25]. In [26,27], the authors proposed a semantic communication system that only transmits a specific task feature. The authors proposed an asynchronous multi-task semantic communication scheme [28], in which the encoder is trained independently using contrastive learning methods, and the decoder executes various communication tasks based on the pre-trained encoder.

### 1.2. Motivation and Contributions

For task-oriented semantic communication, transmitting as few semantic features as possible to achieve the final task can improve communication efficiency. However, most semantic communication models do not optimize the compression rate during design, and only transmit the generated semantic features at a fixed compression rate, greatly reducing communication efficiency. Based on the above analysis, an efficient and robust end-to-end semantic communication framework needs to address three issues:

(1) Reducing the task-independent semantic noise during feature extraction, thereby improving task accuracy;
(2) Reducing the number of training sessions under different channel conditions in the presence of limited computing;
(3) Improving the frequency band utilization of semantic communication systems.

To address the above questions and achieve an efficient and robust semantic communication scheme, we design a task-oriented Scalable Semantic Adaptive Communication (SSAC) framework. To enhance system robustness, the encoder and decoder of the semantic communication system supporting deep neural networks are collaboratively designed and optimized. Simultaneously, by exploring implicit semantic content and incorporating semantic level matching into the encoding and decoding process, we aim to mitigate semantic ambiguity and uncertainty effectively. In summary, our work makes the following contributions:

(1) An Attention Mechanism-based Joint Source Channel Coding (AMJSCC) is designed in order to adapt to the dynamically changing channel conditions in practical communication scenarios, on the basis of task and semantic relationships.
(2) An adaptive channel condition (ACC) module is designed to dynamically adjust the weights and encoding sequence of semantic features, leading to improved robustness in semantic communication and consistent accuracy in classification tasks under channel condition.
(3) In order to generate the multi-scale semantic feature and achieve communication efficiency under bandwidth constraints, a Prediction Scalable Semantic Generator (PSSG) is designed, which includes variable compression ratio, Pre-Net, the optimizer of compression rate, and the loss function. Additionally, the PSSG incorporates attention mechanisms [29] and mask operations [30] to dynamically adapt the length of generated semantic features and could improve system bandwidth utilization without compromising classification performance.

### 1.3. Structure

The rest of this paper is organized as follows: In Section 2, we provide an overview of the proposed SSAC Framework for Task Requirements. Section 3 introduces the AMJSCC, includes a Joint Semantic Channel Coder, and ACC. Section 4 introduces PSSG, includes variable compression ratio, Pre-Net, the optimizer of compression rate, and the loss function. Simulation results are provided in Section 5. Finally, we conclude this work in Section 6.

## 2. Scalable Semantic Adaptive Communication Framework for Task Requirements

**Figure 1:**
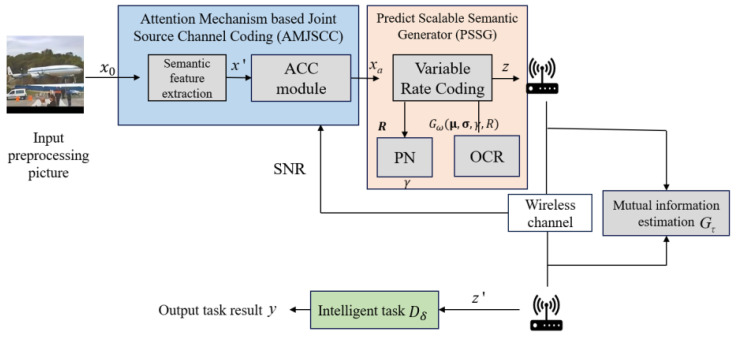
Overview of the proposed SSAC framework.

As shown in Figure 1, this paper proposes a Scalable Semantic Adaptive Communication (SSAC) framework for task requirements. This framework adds an adaptive channel condition module based on semantic relationships–semantic compression (SR-SC) [19], which incorporates channel conditions into the model learning scope through attention mechanism, achieving real-time monitoring and adaptation to changes in channel conditions. This enables the model to dynamically assign weights to key semantic features (SF) at different SNR levels, achieving a low-complexity training mechanism. Due to the detailed explanation and origin of the parameters in Figure 1, shown in Section 3 and Section 4, their definitions are only listed in Table 1.

In order to better adapt to changes in channel conditions, this paper combines semantic feature extraction with importance compression (source coding) and an adaptive channel condition module (channel coding) to form an Attention Mechanism-based Joint Semantic Channel Code (AMJSCC).

In task-oriented semantic communication systems, it is necessary to find the optimal balance between ensuring task performance and minimizing bandwidth. To achieve this balance, this paper designs a Pre-Net prediction module and CR optimizer based on reference. By predicting the performance of image classification tasks at different compression rates, the system model can meet the preset task performance threshold with the minimum compression rate. Subsequently, a Prediction-based Scalable Semantic Generator (PSSG) is used to dynamically adapt the length of generated semantic features during the encoding process based on real-time changes in channel conditions (SNR and bandwidth). This approach enables obtaining scalable semantic information, enhancing flexibility in the semantic communication system, thereby improving resource utilization of channel bandwidth and robustness of semantic communication.

## 3. Attention Mechanic Based Joint Semantic Channel Coding

### 3.1. Joint Semantic Channel Coder

**Figure 2:**
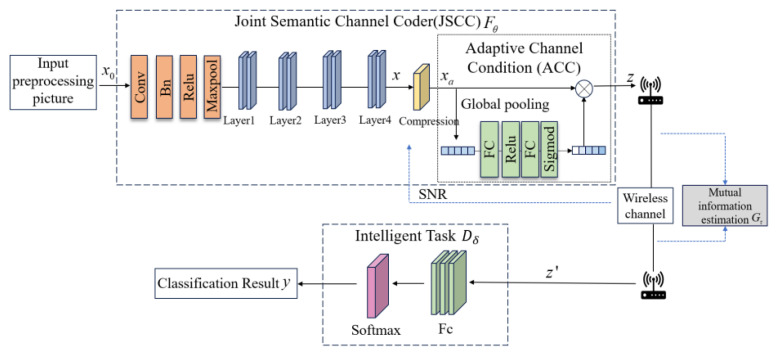
Overview of the AMJSCC module.

The adaptive channel condition module obtains the optimal semantic feature compression rate with the minimum model training cost, and performs image classification with the highest classification accuracy, achieving the goal of adapting to the dynamic changes of the communication channel throughout the entire communication process. As shown in Figure 2, the JSCC encoding method is proposed to facilitate the adjustment of weight allocation for semantic encoding in order to match the SNR conveniently. We use convolution neural networks to extract and compress semantic features from processed input images, and add attention-based modules after compression to dynamically adjust semantic feature weights based on the learned SNR. After receiving semantic information with semantic weights, the receiver enters the intelligent task execution module composed of a fully connected layer and Softmax function to obtain task results.

The entire model consists of a trainable semantic encoder $E_{\varphi }$, an untrained physical channel, and a trainable decoder (intelligent task executor) $D_{\delta }$, where φ and δ represent encoder parameters and decoder parameters, respectively. Specifically, the input processed n dimensional raw data are represented as $x_{0}\in {\mathbb{R}}^{n}$: firstly, a given input image is adaptively encoded to $x^{\prime }$, and extract semantic features with SNR adaptation, then encoded into complex semantic feature codes z of S dimensions, $z\in {\mathbb{C}}^{K}$. The entire encoding process can be represented as follows:

(1) $$z=E_{\varphi }(x_{0},\gamma ,r)$$

where γ represents the channel SNR and $r=S_{z}/S_{x^{\prime }}$ represents the semantic compression rate (CR) of the input image. The obtained signal z will be transmitted through a physical channel and destroyed by Additive Gaussian White Noise (AWGN), and then received by the receiver. The transmission process is formulated as follows:

(2) $$z^{\prime }=\varepsilon z+e$$

where ε is the channel gain coefficient, e is an independent and identically distributed AWGN sample taken from a Gaussian distribution $CN(0,\sigma^{2}I)$, the transmission process is simplified as

(3) $$z^{\prime }=z+e$$

Finally, the received input $z^{\prime }$ is fed into the intelligent task execution module to obtain the task result, which is represented as

(4) $$y=D_{\delta }(z^{\prime })$$

For the extracted semantic features $x^{\prime }$, a gradient-based method [19] is used to extract semantic relationships, and the extracted semantic relationships are ranked in importance. After sorting based on semantic relationship weights, a certain proportion of semantic features are selected for compression. The compression rate r represents the ratio of the actual number of semantic features transmitted for each semantic concept to the total number of semantic features extracted for each semantic concept. So, the actual number of semantic features transmitted can be expressed as $s=\left\lfloor N\cdot r/Q\right\rfloor$, where · represents multiplication, ⌊⬚⌋ represents round down, and r represents the bandwidth compression rate of semantic features.

### 3.2. Adaptive Channel Condition

**Figure 3:**
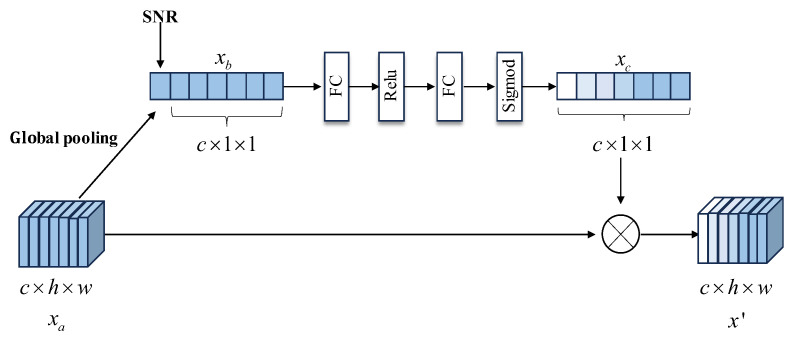
Adaptive channel condition module.

In order to enable the semantic communication model to adapt to a wide range of channel SNR conditions and avoid performance degradation caused by the model’s inability to adapt to dynamically changing SNRs due to training only under fixed SNR conditions, this paper introduces an attention mechanism-based SNR adaptive module. The objective of this approach is to improve the model’s ability to adapt to various SNR environments by incorporating attention mechanisms. The structure of the attention mechanism added to the module is shown in Figure 3. The previously extracted and compressed semantic features $x_{a}$ are used as inputs to the adaptive module:

(5) $$x_{a}=\left[x_{a_{1}},x_{a_{2}},x_{a_{3}},\cdots ,x_{a_{c}}\right]\in {\mathbb{R}}^{c\times h\times w}$$

where c is the number of channels represented, the h and w representing the height and width of semantic features. $x_{a}$ is pooled by the global average pooling, and then the pooled feature *x_b_* is concatenated with the SNR value to obtain context feature SNR.

The contextual information is inputted into a fully connected neural network that primarily consists of two fully connected layers, resulting in a scaling factor $x_{c}$. Finally, the generated scaling factor $x^{\prime }$ is multiplied with the previous semantic features $x_{a}$ to obtain scaling features with different proportional weights:

(6) $$x^{\prime }=\left[{x_{1}}^{\prime },{x_{2}}^{\prime },{x_{3}}^{\prime },\cdots ,{x_{c}}^{\prime }\right]\in {\mathbb{R}}^{c\times h\times w}$$

## 4. Prediction Based on Scalable Semantic Generator

**Figure 4:**
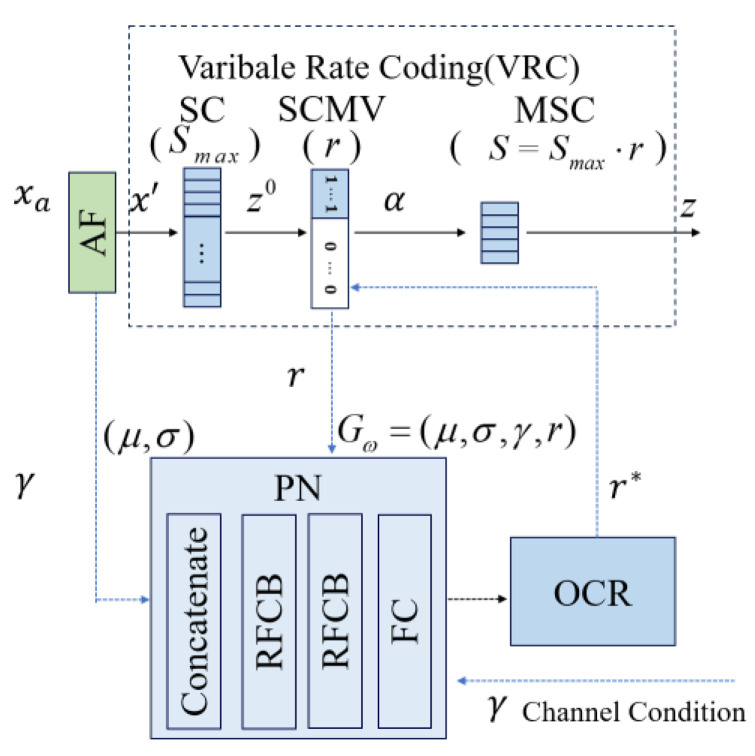
The proposed PSSG scheme.

As depicted in Figure 4, the scalable semantic generator comprises a prediction network, a compression rate optimizer, and a variable compression rate module.

### 4.1. Variable Compression Ratio

The semantic features $x^{\prime }$ obtained from the SNR adaptation module are unfolded to obtain the complex semantic feature $z^{0}\in {\mathbb{C}}^{S_{\max}}$, where $S_{\max}$ represents the maximum length of SC.

Then, use the binary SC mask vector (SCMV) to perform a masking operation, representing $z^{0}$ as $\alpha \in {\left\{0,1\right\}}^{S_{\max}}$, to adjust the length of the semantic features to be transmitted. On the other hand, given a compression ratio value $r\in \left(0,1\right]$, it can be known that the corresponding semantic feature length to be transmitted is $S=\left\lceil r\cdot S_{x^{\prime }}\right\rceil$, which $\left\lceil \cdot \right\rceil$ represents rounding operation. If a complex semantic symbol is represented by two real valued semantic features obtained from the encoder, then the value of the element $\alpha_{i}$ is set to

(7) $$\alpha_{i}=\left\{\begin{array}{l} 1,\;\mathrm{if}\;i\leq S\\ 0,\;\mathrm{otherwise} \end{array}\right.$$

The SC mask operation can be expressed as

(8) $$z=z^{0}\cdot \alpha$$

where ⋅ represents dot product operation. When $\alpha_{i}=1$, the corresponding semantic features were selected for transmission, which means that the first element of SC was selected as the mask SC (MSC) and transmitted to the receiver.

In this paper, the semantic features $x^{\prime }$ obtained from the SNR adaptive module are unfolded to obtain complex semantic features $z^{0}$ that are actually two-dimensional data with a shape of c×(h×w), where c represents the feature channel, h and w represent the feature height and width, respectively. In this case, SCMV can be a binary vector with a length c. Given the encoding length S, the semantic features of the previous S channels will be selected for transmission, and the remaining channels will be discarded. After receiving semantic features, the receiver only classifies based on these semantic features to obtain classification results.

### 4.2. Pre-Net

**Figure 5:**
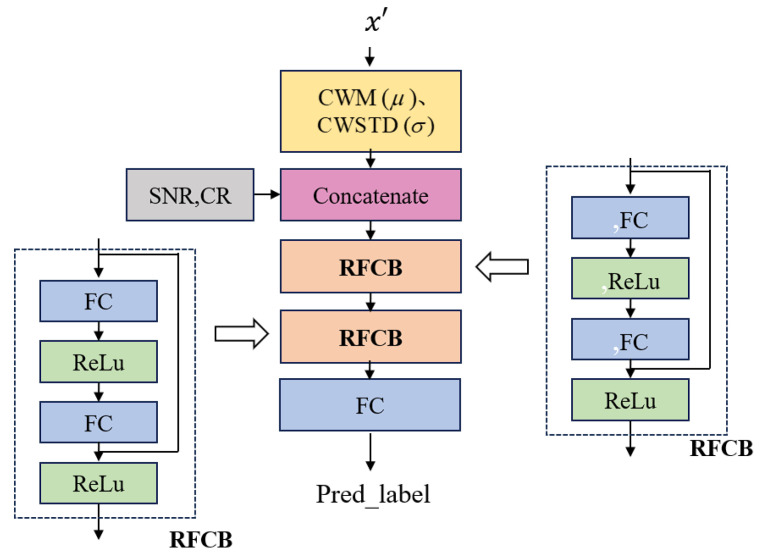
Pre-Net scheme.

The architecture of the Pre-Net network model is shown in Figure 5, which consists of two Residual Fully Connected Blocks (RFCBs), Concatenate, and Full Connection (FC) layer. $x^{\prime }$ is the semantic compression on the original input image, which has the most relevant image feature to the task, such as edges, color changes, and textures. If using raw image data to predict classification results, a larger model will be required to understand the complex image content, and the computational complexity will greatly increase. In order to further reduce complexity and maintain dimensional stability, the Channel Wise Mean (CWM) or Channel Wise Standard Deviation (CWSTD) used in the Principal Component Analysis (PCA) method [30] are referred to $x^{\prime }$ as semantic content features to predict image classification results. In the model, CWM and CWSTD are represented by μ and σ, respectively. It is assumed that the shape of $x^{\prime }$ has 512 channels, with each channel having a width and height of 7. Therefore, the dimensions of both μ and σ are 512, resulting in a total of 1024 semantic content feature values. The Concatenate is to learn the relationship between image classification results and image content.

The main function of RFCB is to help solve two problems: ① gradient vanishing and ② gradient explosion, that may be encountered during the training process of deep neural networks. The fully connected layer is responsible for learning nonlinear combinations between features. By combining them, information and gradients can be effectively transmitted between different layers, thereby improving the learning ability and performance of the network. This structure is particularly effective in handling complex data and tasks, such as image recognition, speech processing, and other fields. By utilizing these two RFCBs, the model can effectively extract representative information for predicting image classification results, and can use the last FC layer to obtain the predicted classification results. Overall, the complexity of Pre-Net is much lower than that of the VDJSCC model, and its efficiency is much higher.

### 4.3. The Optimizer of Compression Rate

By using the trained VDJSCC and Pre-Net models, it is possible to minimize the CR value under certain classification accuracy constraints to improve bandwidth efficiency. In this problem, the classification accuracy constraint is to ensure that the classification accuracy of the semantic features received by the receiving end is not lower than the target threshold ${\mathrm{Acc}}_{th}$. According to semantic content in the image to be classified, the optimization of CR can be categorized into two types: data level and instance level. Specifically, data-level CR optimization can solely rely on the VDJSCC model without considering image content, while instance-level CR optimization requires both Pre-Net prediction model and VDJSCC model to consider the image content.

Data-level CR optimization: The VDJSCC model makes the compression rate of the overall semantic communication system controllable, which means that it only needs to be trained once to achieve good classification performance at multiple levels of compression rates. During testing, the number of SNR levels is $N_{SNR}$ and the level of the compression rate is $N_{CR}$, a classification accuracy of size $N_{SNR}\times N_{CR}$ under the VDJSCC model can be obtained as U(γ,r). The process of ensuring that the classification accuracy of semantic features received by the receiver is not lower than the target value ${\mathrm{Acc}}_{th}$ can be expressed as

(9) $$\min r\;s.t.U(\gamma ,r)\geq {\mathrm{Acc}}_{th},0\leq r\leq 1$$

To obtain the optimal CR value *r* when the classification accuracy is not lower than the target value ${\mathrm{Acc}}_{th}$, it is only necessary to find it through an exhaustive search in the table U(γ,r). This method can obtain the optimized CR value without considering the content of the image to be classified.

### 4.4. The Loss Function

The comparative logarithmic upper bound (CLUB) estimation method aims to solve the problem of minimizing mutual information (MI) in high-dimensional space. The vCLUB method uses CLUB to estimate the MI between two random variables. The core of this method lies in utilizing the idea of contrastive learning to estimate the upper bound of mutual information by comparing the logarithmic ratio of the conditional probabilities of positive and negative sample pairs. The definition of vCLUB is

(10) $$I_{vCLUB}(x;y):=E_{p(x,y)}[\log(q_{\theta }(y\;|\;x)]-E_{p(x)}E_{p(y)}[\log(q_{\theta }(y\;|\;x)]$$

where the variational distribution $q_{\theta }(y\;|\;x)$ is used to approximate the true conditional distribution p(y | x), and the fitting distribution of the neural network with parameters θ.

The training objective of the entire model of attention-based SNR adaptive semantic communication proposed in this paper is to simultaneously minimize cross entropy and maximize mutual information between the original data and classification results. Therefore, its loss function can be expressed in the following form:

(11) $$L_{\theta }=-\sum_{c=1}^{Q}y\log(p_{c})-\beta I_{\mathrm{vCLUB}}$$

where the weighting factor β between minimizing cross entropy and mutual information has a range of values [0,1]. By minimizing cross entropy during model training and maximizing mutual information to ensure high-precision intelligent task execution, this balancing strategy not only enhances the system’s intelligent processing capability, but also significantly reduces the bandwidth requirement for data transmission, which is the key to achieving efficient and energy-saving communication.

## 5. Experiments

In order to better evaluate the performance advantages of the proposed model framework in image classification tasks, this paper selects traditional communication methods (images are encoded in JPEG and transmitted to the decoding terminal for classification) and SC-AITs [19] (semantic relationships and semantic compression) as baselines to compare image classification performance under different SNRs and semantic feature compression rates.

### 5.1. Experimental Data and Settings

This paper is based on the Linux operating system to design a simulation platform, with specific parameters shown in Table 2.

The datasets used in this experiment are the STL-10 public dataset and the CIFAR-10 public dataset, both of which contain 10 different categories of images. The difference is that they contain different categories and image sizes. These two datasets cover some common objects and scenes, providing a good benchmark for multi-class classification problems. The performance of the model in handling multi-class image classification tasks can be evaluated by using these two datasets. During training, the image is first expanded and randomly cropped to reduce its size 224×224. Next, the cropped image is fed into the training network.

When training the network, interactively train the mutual information estimation network and semantic classification network. First, train the mutual information network, and then train the semantic communication network while keeping the parameters of the mutual information network unchanged. Next, while keeping the parameters of the semantic communication network unchanged, train the mutual information network again. Repeat this cycle alternately until the conditions for ending the training are met. Set the number of iterations loop number to 10, epoch to 30, batch size to 64, use the Stochastic Gradient Descent (SGD) optimizer, set the learning rate to 0.001, and the loss function to be the difference between the classification loss function and the mutual information loss function. Choose the cross entropy loss function as the classification loss function. The specific network training parameters are shown in Table 3.

For the convenience of comparison and analysis of experimental results, this article uses Table 4 to conduct experiments on various communication frameworks. Ultimately, accuracy (ACC) will be used as a comparative evaluation metric, defined as the ratio of the number of correctly classified images to the number of images to be classified.

### 5.2. The Analysis and Comparison of Experimental Results

#### 5.2.1. Subsection

In this experiment, the SR-SC [19] model was multiple trained under different SNR values (such as: 0 dB, 5 dB, 10 dB, 15 dB, 20 dB, and 25 dB). However, our proposed algorithm AMJSCC only takes one training model under random SNR conditions (such as [0, 25] dB), which is the most advantageous for our model.

**Figure 6:**
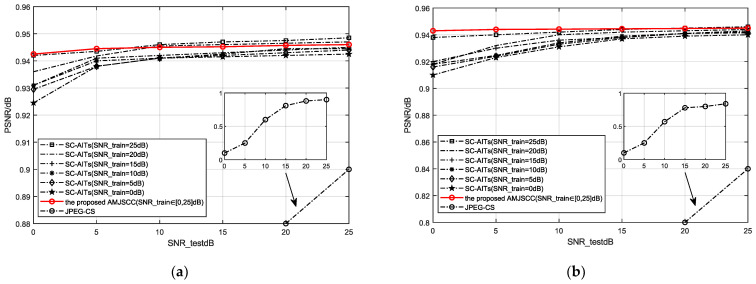
Classification performance of different methods. (**a**) STL-10 test images. (**b**) CIFAR-10 test images.

Figure 6 shows the classification performance comparison of three communication methods in the STL-10 dataset and CIFAR-10 dataset, where the solid line represents the classification performance of AMJSCC proposed in this paper, and the dashed line represents the final classification performance of JPEG-CS [19] and SC-AITs [19]. It should be noted that the framework presented in this article has not been subjected to semantic importance ranking and compression, i.e., all semantic features have been transmitted, and like the comparative literature, the scalability of compression has not been considered.

In Figure 6, due to the low classification accuracy obtained by the traditional method JPEG-CS [19] at low SNRs, it is not possible to display all of them on one image. Therefore, local enlarged supplementary images were placed. As shown in the figure, the classification accuracy of the AMJSCC framework proposed in this paper is significantly better than the traditional JPEG-CS [19] method in image classification tasks. In low SNR environments (such as 0 dB), the classification accuracy of the JPEG-CS [19] method is less than 20%, making it impossible to complete the classification task. The classification accuracy of the algorithm proposed in this article is over 94%, which can effectively complete the classification task. This result fully confirms that in the field of communication for image classification tasks, the attention-based semantic communication AMJSCC framework has significant performance advantages compared to traditional image compression communication methods, especially in low SNR environments.

From Figure 6, it can be seen that the SC-AIT [19] method has poor applicability in training models under different SNR conditions: the classification performance of low SNR training models is lower than that of high SNR training models. For example, the model trained on the STL-10 dataset with a fixed SNR of 0 dB has a classification accuracy ranging from 92.4% to 94%, while the model trained with a fixed SNR of 25 dB has a classification accuracy ranging from 94% to 95%. Therefore, the model trained multiple times by SC-AITs [19] has a classification accuracy between 92% and 95%, which is a large range and unstable classification performance.

The AMJSCC framework proposed in this article fully considers different SNR conditions from low to high during training, and can achieve optimal classification performance (classification accuracy of about 94.3%) regardless of changes in SNR conditions during testing. It is more stable than the SC-AIT [19] method and comparable to the optimal model performance of SC-AITs [19]. For example, on the CIFAR-10 dataset, the AMJSCC method proposed in this paper achieved a classification accuracy of 94.3% when testing an SNR of 0 dB, which is only 0.6% different from the optimal testing classification accuracy of 93.7% obtained by the SC-AIT method [19] trained at a fixed SNR of 25 dB. In addition, for the STL-10 dataset, the difference between the highest and lowest classification accuracy of the JPEG-CS method and the SR-SC method was about 80% and 1.7%, respectively, within the tested SNR range [0,25] dB. However, the AMJSCC method proposed in this paper only had a difference of about 0.3% between the highest and lowest classification accuracies. This highlights its strong robustness to signal quality fluctuations.

#### 5.2.2. The PSSG Result Analysis

(1) Performance Analysis of Pre-Net Prediction

**Figure 7:**
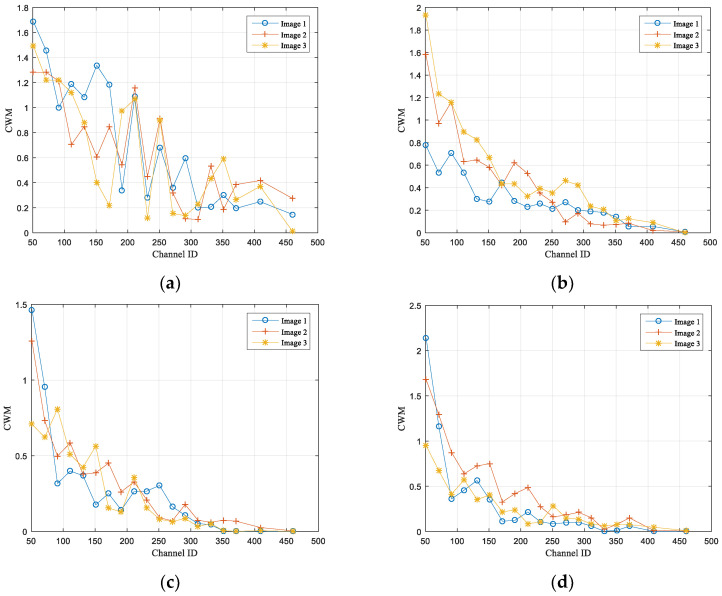
CWM characteristics: (**a**) airplane, (**b**) bird, (**c**) car, (**d**) cat.

**Figure 8:**
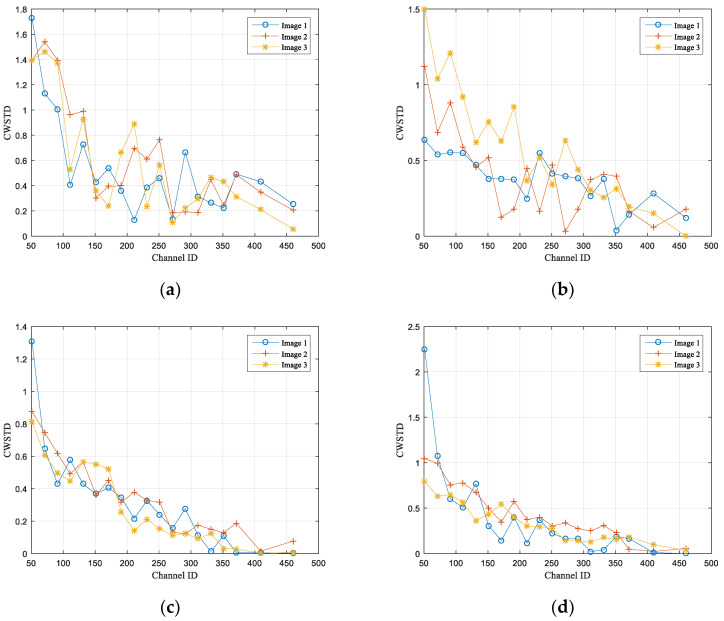
CDSTD characteristics: (**a**) airplane, (**b**) bird, (**c**) car, (**d**) cat.

The CWM and CWSTD in the Pre-Net model mentioned in this article are respectively the channel mean and channel standard deviation of semantic features $x^{\prime }$ obtained after semantic feature extraction and channel adaptation module encoding. They are selected as semantic content features based on principal component characteristics to predict image classification results. For the training and validation images of the STL10 dataset, this experiment follows the steps in Section 4.2 to expand and crop the images into sizes 3×224×224 and input them into the network. The semantic feature $x^{\prime }$ size extracted by the SNR adaptive module AF is changed to 512×7×7, where 512 is the number of channels for the feature. Since the CR is randomly generated within the range of [0.1, 0.9] during model training, the number of semantic feature MSC channels after SC masking operation is within the range of [51, 460]. In order to test the feature characteristics of CWM and CWSTD, three test images, Image 1, Image 2, and Image 3, were randomly selected for each category of the STL-10 test dataset. Their values on channel [51, 460] were calculated separately, and the SNR condition was set to 25 dB during testing. Figure 7 and Figure 8, respectively, show the characteristics of CWM and CWSTD, where (a), (b), (c), and (d) show the image results selected from four categories in the STL-10 dataset.

From the figures, it can be seen that the CWM and CWSTD features of randomly selected images show a gradually decreasing trend from low to high channel numbers, indicating that after learning, the semantic features of low and high channels have more information compared to each other. This trend is due to the use of SC masking operations in the model to filter out the semantic features of a portion of the channels, while retaining the semantic features of the previous S channels in each transmission. This allows the model to automatically learn to compress more important content information into channels with relatively higher positions during training.

The above results indicate that after model training and learning, more information of the image is contained in the low channels of semantic features. Based on the actual channel situation, some channel features with more information can be selected for compression transmission, and the optimal compression rate can be found by predicting the accuracy of image classification, providing a basis for semantic scalability.

**Figure 9:**
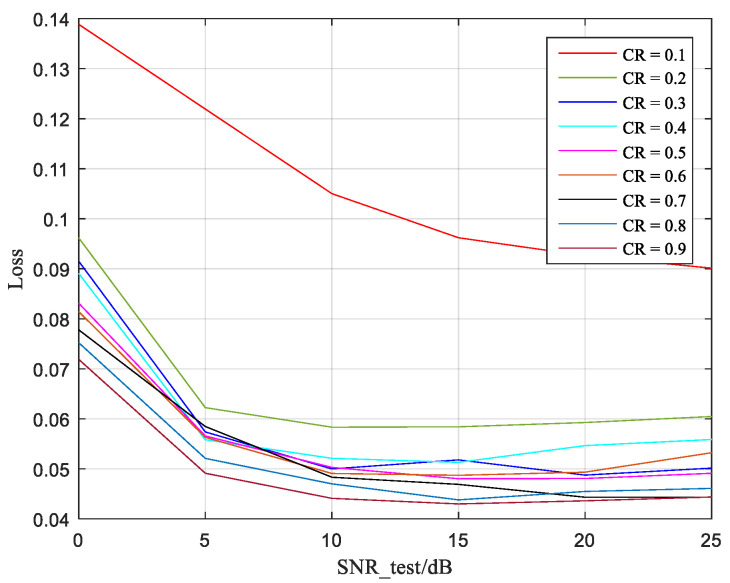
Loss of Pre-Net predicted.

From Figure 9, it can be observed that at extremely low SNR, such as 0 dB, the Pre-Net model predicts relatively high losses. This is because at very low SNR, semantic features are relatively more damaged by noise in the AWGN channel, resulting in significant errors between the predicted classification results and the actual classification results. As the SNR increases, the loss results generally show a gradually decreasing trend, but in high SNR situations, the loss tends to flatten out as the SNR continues to increase. This is because when the SNR is high enough, the classification performance of the PSSG model trained in this paper gradually converges to the upper limit. Even if the SNR continues to increase, the actual classification results of the PSSG model will not change much.

Overall, as the SNR increases, the predictive performance of the Pre-Net prediction model proposed in this paper improves, and the prediction loss performance can even approach 0.04, which is obtained under moderate SNR and verifies the predictive effectiveness of the Pre-Net model.

(2) Compression ratio optimization OCR results

In order to analyze the performance of the compression rate optimizer proposed in this article, under certain classification accuracy constraints, 18 optimized CR values r∈[0.1:0.05:0.9] were used to test the minimum compression rate CR value of the model, which is the minimum compression rate that can be achieved when ensuring that the received semantic feature classification accuracy is not lower than the target ${\mathrm{Acc}}_{th}$.

**Figure 10:**
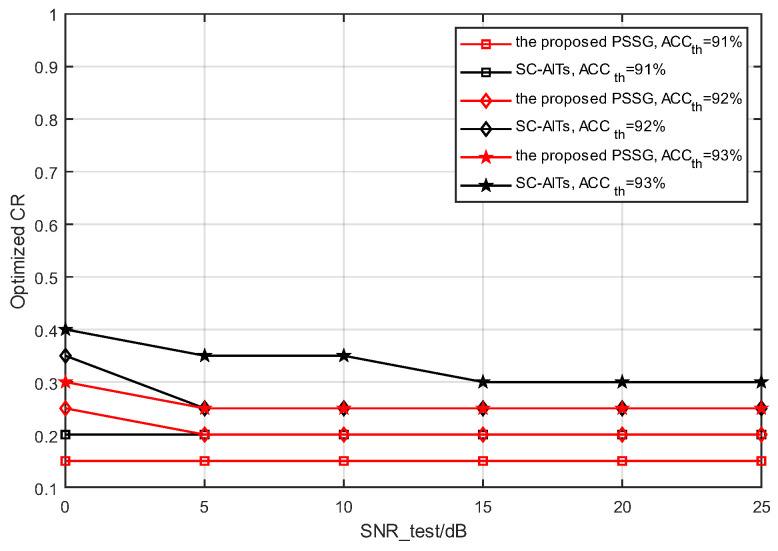
The minimum CR values for different methods.

As shown in Figure 10, under the same classification accuracy constraint, compared with the SC-AIT [19] method, the PSSG algorithm proposed in this paper can achieve lower CR values, which is consistent with the classification accuracy performance comparison between the PSSG algorithm and SC-AITs [19] shown in Figure 6. In addition, for the PSSG algorithm in this article, even with only one model in testing, it can provide 18 CR level choices, while SC-AITs [19] requires more training models to provide corresponding CR choices. The above results indicate that the PSSG algorithm proposed in this paper can achieve the same or even higher classification accuracy with fewer semantic features, greatly improving overall communication efficiency, and reducing computational and deployment costs because the model only needs to be trained once to meet the requirements.

Based on the above analysis, the PSSG framework proposed in this article can adapt to various compression rates under light training, and can also achieve relatively satisfactory classification accuracy under low-compression rate conditions. On the other hand, the PSSG model proposed in this article can transmit the minimum number of semantic features to achieve the expected classification results, thereby improving communication efficiency.

#### 5.2.3. The Analysis of Classification Performance of SSAC

**Figure 11:**
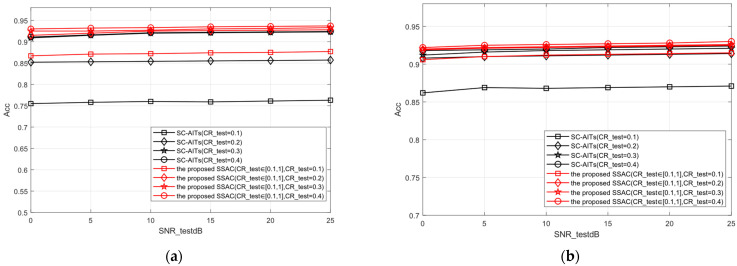
Comparison of classification accuracy performance of different methods. (**a**) STL-10 test images, (**b**) CIFAR-10 test images.

In order to compare the classification performance of scalable semantics (i.e., semantic features with different compression rates), compression rates were set to 0.1, 0.2, 0.3, and 0.4, and four corresponding SC-AIT [19] models were trained. Meanwhile, the SSAC model of this paper was trained under different compression ratios (CR = [0.1:0.9]). For all SSAC and SC-AIT [19] models, the SNR is obtained from [0,25]dB randomly. In order to fully compare the impact of semantic feature compression rate changes on classification characteristics, except for the SC mask, the architecture of the encoder and decoder in SSAC and SC-AITs [19] is the same, and the importance ranking of semantic features is performed before transmitting them. At this point, the classification performance of each is studied based solely on the different lengths of semantic features (i.e., different compression rates), as shown in Figure 11.

The comparative analysis and conclusion of the classification accuracy between the SSAC scheme and SC-AIT [19] scheme under the same and different compression ratios are as follows:

(1) Whether under high or low SNR conditions, the SSAC proposed in this paper can achieve classification accuracy similar to SC-AITs [19] at the same compression rate (such as CR = 0.3). This indicates that SSAC can maintain good stability and robustness in high dynamic range channels.
(2) At lower compression rates (such as CR = 0.1), the SSAC method proposed in this paper significantly outperforms the SC-AIT [19] method in terms of classification accuracy after compression. Taking the STL-10 dataset as an example, when using SC mask for semantic feature compression with a compression rate of 0.1, the classification accuracy of the SSAC method is about 11% higher than SC-AITs [19]. This indicates that the SSAC classification performance proposed in this paper is better at lower compression rates, which means it has the advantage of preserving important features of classification tasks.
(3) In the case of a single model, the overall performance of the proposed SSAC model is equivalent to or even better than that of multiple models trained by SC-AITs [19] using different compression rates. This indicates that SSAC can achieve scalable semantic features by adapting to different compression requirements through a single training session, thereby helping to reduce the resource consumption of training and deployment. This is highly attractive for application scenarios that require the deployment of efficient semantic communication systems in resource constrained environments.

#### 5.2.4. The Analysis of the Complexity of SSAC

This experiment conducted a detailed comparison of the computing complexity with other methods based on the number of floating-point operations (FLOPs), model parameter count (Params), and memory usage metrics. The results are shown in Table 5.

**Figure 12:**
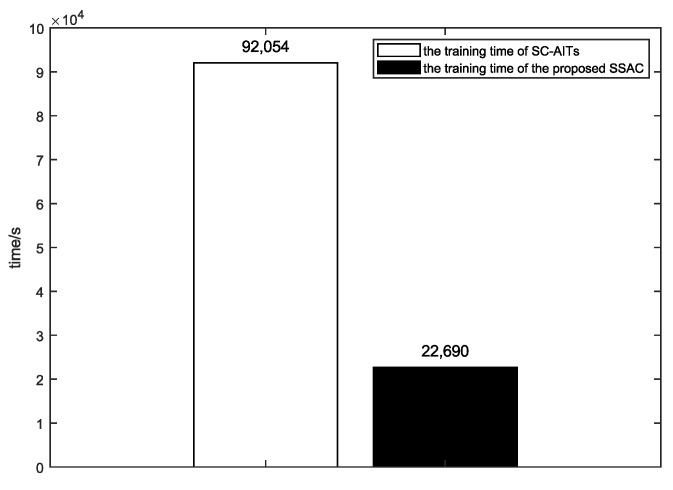
Comparison of training time of different methods.

The experiment recorded the training time required for the comparison method and the proposed method to achieve the highest image classification accuracy under the condition of SNR_test = [0, 5, 10, 15, 20, 25], which are shown in Figure 12. The results show that the training time required for the proposed method is much less than that of the comparison method. This is because in order to obtain the highest image classification accuracy under the corresponding SNR conditions, the comparison method must be trained multiple times at a fixed SNR, while the method proposed in this paper only needs to be trained once.

## 6. Conclusions

In this paper, we investigated a task-oriented SSAC framework for the WSNs. Our contribution was to introduce the generation of scalable semantics, which provides a more reasonable evaluation of semantic-level aspects during the training process for different channel conditions.

Moreover, we modified the Joint Semantic Channel Coder by adding the Attention Mechanism, which can adapt to the channel condition module obtaining the optimal semantic feature compression rate with the minimum model training cost and performs image classification with the highest classification accuracy, achieving the goal of adapting to the dynamic changes of the communication channel throughout the entire communication process. On the other hand, we have modified the traditional formal approach with a practical wireless channel and proposed the Scalable Semantic Prediction Generator approach, which allows us to comprehensively exploit the impact of the channel on the transmission of semantic information. Further, we designed training procedures for SSAC, which achieved a good trade-off between preserving semantic information and retaining intricate details.

Finally, we conducted simulations under various conditions, including different bandwidth compression ratios, SNRs, and different model configurations, to demonstrate the effectiveness and robustness of the proposed approaches. From the results of the experiment, the SSAC framework has advantages such as stability, efficiency, universality, robustness, and low training complexity, effectively improving the performance and bandwidth utilization of semantic communication systems.

## Figures and Tables

**Table 1:** The parameter definitions for Figure 1.

*x*_0_	The input image	*z*	The transmitted SF	σ	The Channel Wise Standard Deviation
*x′*	The extracted SF	*z′*	The received SF	γ	The compression ratio value
*x_a_*	The compressed SF	μ	The Channel Wise Mean	*R*	The bandwidth compression rate of SF

**Table 2:** The detail description of hardware and dataset in the exprimental.

Hardware and Dataset	Description
CPU	Intel(R) Core(TM) i7-9700
GPU	NVIDIA GeForce RTX 4060 Ti (x2)
Memory capacity	32 GB
GPU driver version	535.146.02
CUDA version	11.7
Operating system	Ubuntu 18.04.6 LTS
Network conditions	1000 Mbps
Dataset 1	Self-Taught Learning 10 (STL-10)
Dataset 1 size	Training set: 5000 images; Test set: 8000 images
Dataset 1 image Size	96×96
Dataset 2	Canadian Institute for Advanced Research 10 (CIFAR-10)
Dataset 2 size	Training set: 5000 images; Test set: 10,000 images
Dataset 2 image size	32×32

**Table 3:** Network hyper parameter settings of SSAC training algorithm for image classification tasks.

The Name of Parameter	The Value of Parameter
Loop_num	10
Epochs	30
Batch size	64
Weight decay	SGD
Learning rate (lr)	0.001
Momentum factor (Momentum)	0.9
Weight_decay	0
Learning rate decay period (step_size)	7
Multiplicative factor for learning rate decay	0.1
β	0.0001
SNR	[0, 25]

**Table 4:** Experimental process description of communication frameworks for image classification tasks.

Communication Methods	Experimental Process of Communication Framework
JPEG-CS [19]	1. First, perform traditional JPEG compression encoding; 2. Adopting LDPC encoding with a code rate of 2/3 and 16QAM modulation; 3. And then transmitted through the channel to the receiving end; 4. The receiving end decodes and restores the image; 5. Then classify and process the restored images.
SC-AITs [19]	1. Firstly, the image is subjected to a joint semantic encoder to extract semantic feaures; 2. Transmitting semantic features to the receiving end under fixed SNR; 3. The receiving end performs classification tasks based on the received features.
Our SSAC	1. Adjust the semantic feature weights of the extracted semantic features through a SNR adaptation module; 2. Send the sorted semantic features to the semantic channel joint encoder (AMJSCC) for processing, achieving the semantic encoding characteristics of adaptive channel conditions; 3. Then it is fed into a prediction based scalable semantic generator (PSSG) to achieve scalable semantic features with variable compression rates; 4. Finally, under channel conditions within the SNR range, semantic features are transmitted to the receiving end to complete the classification task.

**Table 5:** Experimental results of computing complexity.

Model	FLOPs	Params	Memory
SC-AITs [19]	1.73 G	11.64 M	65.63 MB
The proposed SSAC	1.63 G	11.09 M	65.24 MB

## Data Availability

The datasets generated and/or analyzed during this study may be obtained from the corresponding author upon reasonable request.

## References

[1] Liu X., Huang Z., Zhang Y., Jia Y., Wen W. (2024). CNN and Attention-Based Joint Source Channel Coding for Semantic Communications in WSNs. Sensors.

[2] Sharma B., Koundal D., Ramadan R.A., Corchado J.M. (2023). Emerging Sensor Communication Network-Based AI/ML Driven Intelligent IoT. Sensors.

[3] Shi Y., Zhou Y., Wen D., Wu Y., Jiang C., Letaief K.B. (2023). Task-Oriented Communications for 6G: Vision, Principles, and Technologies. IEEE Wirel. Commun..

[4] Yang W., Du H., Liew Z.Q., Lim W.Y.B., Xiong Z., Niyato D., Chi X., Shen X.S., Miao C. (2022). Semantic communications for future internet: Fundamentals, applications, and challenges. IEEE Commun. Surv. Tutorials.

[5] Fu Q., Xie H., Qin Z., Slabaugh G., Tao X. (2023). Vector Quantized Semantic Communication System. IEEE Wirel. Commun. Lett..

[6] Luo X., Gao R., Chen H.H., Chen S., Guo Q., Suganthan P.N. (2024). Suganthan. Multimodal and Multiuser Semantic Communications for Channel-Level Information Fusion. IEEE Wirel. Commun. Lett..

[7] Xie H., Qin Z., Li G.Y., Juang B.H. (2020). Deep learning based semantic communications: An initial investigation. Proceedings of the GLOBECOM 2020-2020 IEEE Global Communications Conference.

[8] Xie H., Qin Z., Li G.Y., Juang B.H. (2021). Deep learning enabled semantic communication systems. IEEE Trans. Signal Process..

[9] Jiang P., Wen C.K., Jin S., Li G.Y. (2023). Wireless semantic communications for video conferencing. IEEE J. Sel. Areas Commun..

[10] Wang S., Dai J., Liang Z., Niu K., Si Z., Dong C., Qin X., Zhang P. (2023). Wireless deep video semantic transmission. IEEE J. Sel. Areas Commun..

[11] Lan Q., Wen D., Zhang Z., Zeng Q., Chen X., Popovski P., Huang K. (2021). What is semantic communication? A view on conveying meaning in the era of machine intelligence. J. Commun. Inf. Netw..

[12] Lokumarambage M.U., Gowrisetty V.S.S., Rezaei H., Sivalingam T., Rajatheva N., Fernando A. (2023). Wireless end-to-end image transmission system using semantic communications. IEEE Access.

[13] Güler B., Yener A., Swami A. (2018). The semantic communication game. IEEE Trans. Cogn. Commun. Netw..

[14] Fu Y., Cheng W., Zhang W. (2023). Content-aware semantic communication for goal-oriented wireless communications. Proceedings of the IEEE INFOCOM 2023-IEEE Conference on Computer Communications Workshops (INFOCOM WKSHPS).

[15] Luo X., Chen H.H., Guo Q. (2022). Semantic communications: Overview, open issues, and future research directions. IEEE Wirel. Commun..

[16] Dinh N.T., Van T.T., Le T.M. (2022). Semantic relationship-based image retrieval using kd-tree structure. Proceedings of the Asian Conference on Intelligent Information and Database Systems.

[17] Kang X., Song B., Guo J., Qin Z., Yu F.R. (2022). Task-oriented image transmission for scene classification in unmanned aerial systems. IEEE Trans. Commun..

[18] Pan Q., Tong H., Lv J., Luo T., Zhang Z., Yin C., Li J. (2023). Image segmentation semantic communication over internet of vehicles. Proceedings of the 2023 IEEE Wireless Communications and Networking Conference (WCNC).

[19] Yang Y., Guo C., Liu F., Sun L., Liu C., Sun Q. (2023). Semantic Communications With Artificial Intelligence Tasks: Reducing Bandwidth Requirements and Improving Artificial Intelligence Task Performance. IEEE Ind. Electron. Mag..

[20] Mingkai C., Minghao L., Zhe Z., Zhiping X., Lei W. (2024). Task-oriented semantic communication with foundation models. China Commun..

[21] Tian Z., Wang W., Zhou K., Song X., Shen Y., Liu S. (2024). Weighted Pseudo-Labels and Bounding Boxes for Semisupervised SAR Target Detection. IEEE J. Sel. Top. Appl. Earth Obs. Remote. Sens..

[22] Deng J., Wang W., Zhang H., Zhang T., Zhang J. (2024). PolSAR Ship Detection Based on Superpixel-Level Contrast Enhancement. IEEE Geosci. Remote. Sens. Lett..

[23] Gündüz D., Qin Z., Aguerri I.E., Dhillon H.S., Yang Z., Yener A., Wong K.K., Chae C.B. (2023). Beyond transmitting bits: Context, semantics, and task-oriented communications. IEEE J. Sel. Areas Commun..

[24] Hu Q., Zhang G., Qin Z., Cai Y., Yu G., Li G.Y. (2022). Robust semantic communications against semantic noise. Proceedings of the 2022 IEEE 96th Vehicular Technology Conference (VTC2022-Fall).

[25] Sun Z., Liu F., Yang Y., Tong W., Guo C. (2023). Multi-task semantic communications: An extended rate-distortion theory based scheme. Proceedings of the 2023 IEEE International Conference on Communications Workshops (ICC Workshops).

[26] Zhang G., Hu Q., Qin Z., Cai Y., Yu G. (2022). A unified multi-task semantic communication system with domain adaptation. Proceedings of the GLOBECOM 2022–2022 IEEE Global Communications Conference.

[27] Zhang G., Hu Q., Qin Z., Cai Y., Yu G., Tao X. (2024). A unified multi-task semantic communication system for multimodal data. IEEE Trans. Commun..

[28] Tian Z., Vo H., Zhang C., Min G., Yu S. (2023). An asynchronous multi-task semantic communication method. IEEE Netw..

[29] Zhang W., Zhang H., Ma H., Shao H., Wang N., Leung V.C. (2023). Predictive and adaptive deep coding for wireless image transmission in semantic communication. IEEE Trans. Wirel. Commun..

[30] Greenacre M., Groenen P.J., Hastie T., d’Enza A.I., Markos A., Tuzhilina E. (2022). Principal component analysis. Nat. Rev. Methods Primers.

